# Illness Cognitions in ALS: New Insights Into Clinical Management of Behavioural Symptoms

**DOI:** 10.3389/fneur.2021.740693

**Published:** 2021-09-24

**Authors:** Jashelle Caga, Emma Devenney, William Huynh, Margaret C. Zoing, Rebekah M. Ahmed, Matthew C. Kiernan

**Affiliations:** ^1^Brain & Mind Centre, University of Sydney, Camperdown, NSW, Australia; ^2^Sydney Medical School, University of Sydney, Camperdown, NSW, Australia; ^3^Prince of Wales Clinical School, University of NSW, Sydney, NSW, Australia; ^4^Royal Prince Alfred Hospital, Sydney, NSW, Australia

**Keywords:** amyotrophic lateral sclerosis (ALS), illness perceptions, behavioural symptoms, patient decision-making, adherence - compliance - persistance, behavioural intervention, health care delivery

## Abstract

Timely management of frontotemporal dysfunction associated with amyotrophic lateral sclerosis (ALS) has important prognostic and therapeutic implications. However, there remains a paucity of research on best practise recommendations to guide the development of interventions for cognitive and behavioural symptoms as part of ALS care. Accordingly, a focus on illness perceptions may provide a preliminary framework for managing cognitive and behavioural symptoms. The aim of the present study was to explore the nature of illness perceptions among ALS patients with cognitive and behavioural symptoms. A total of 39 patients were recruited from a specialised ALS clinic. Factor analysis showed three independent and clinically interpretable factors corresponding to “cognitive and emotion related ALS perceptions,” “cognitive- specific ALS perceptions” and “ALS coherence”. Of these factors, greater perceived cognitive and emotional impacts of ALS were associated with an approximate 4-fold increased risk of behavioural changes (*p* < 0.05). Greater perceived cognitive and emotional impacts of ALS was also associated with more rapid disease progression (*p* < 0.001). As such, timely provision of intervention addressing perceptions about the impact of ALS on functioning as well as associated emotional distress may optimise clinical management of cognitive and behavioural symptoms of ALS.

## Introduction

Amyotrophic lateral sclerosis (ALS) is a multisystem neurodegenerative disorder which includes a broad spectrum of non-motor symptoms that can dominate the clinical picture (1). The incidence of cognitive and behavioural changes in ALS may vary between 10–75%, with up to 14% of patients meeting the criteria for a diagnosis of concomitant frontotemporal dementia (FTD) based on population studies (2, 3). In addition to significant cognitive problems, ALS patients may experience behavioural symptoms that include perseveration, disinhibition and most commonly apathy (4, 5).

The challenge of providing appropriate and effective care for ALS patients primarily falls to specialised ALS clinics. Symptom management including timely referrals to respiratory and palliative care and optimising quality of life remain the mainstays of treatment (6), requiring an interdisciplinary care approach often involving multiple physicians and allied health professionals. Compared to community-based care, specialised multidisciplinary ALS clinical services have consistently been associated with more positive outcomes including improved prognosis (7–9) and quality of life (10). ALS patients attending specialised multidisciplinary ALS clinics have an increased utilisation of Riluzole and interventions such as ventilatory support and gastrostomy, resulting in fewer hospitalisations (11).

However, studies to date have primarily involved ALS patients without assessment of cognitive and behavioural symptoms and therefore the potential role of specialised multidisciplinary ALS care for patients presenting with such symptoms remains largely unknown. A major barrier may relate to the relative paucity of research that directly examines factors influencing illness-related behaviour among ALS patients with cognitive and behavioural symptoms (12–15). It is unlikely that ALS patients have complete lack of awareness of their condition, but clearly their insight into specific symptoms and how it affects their well-being differs from their caregivers. Indeed, ALS patients with subtle behavioural changes have been shown to have spared insight compared to ALS-FTD patients who lack insight into their behavioural symptoms (16).

**Figure 1:**
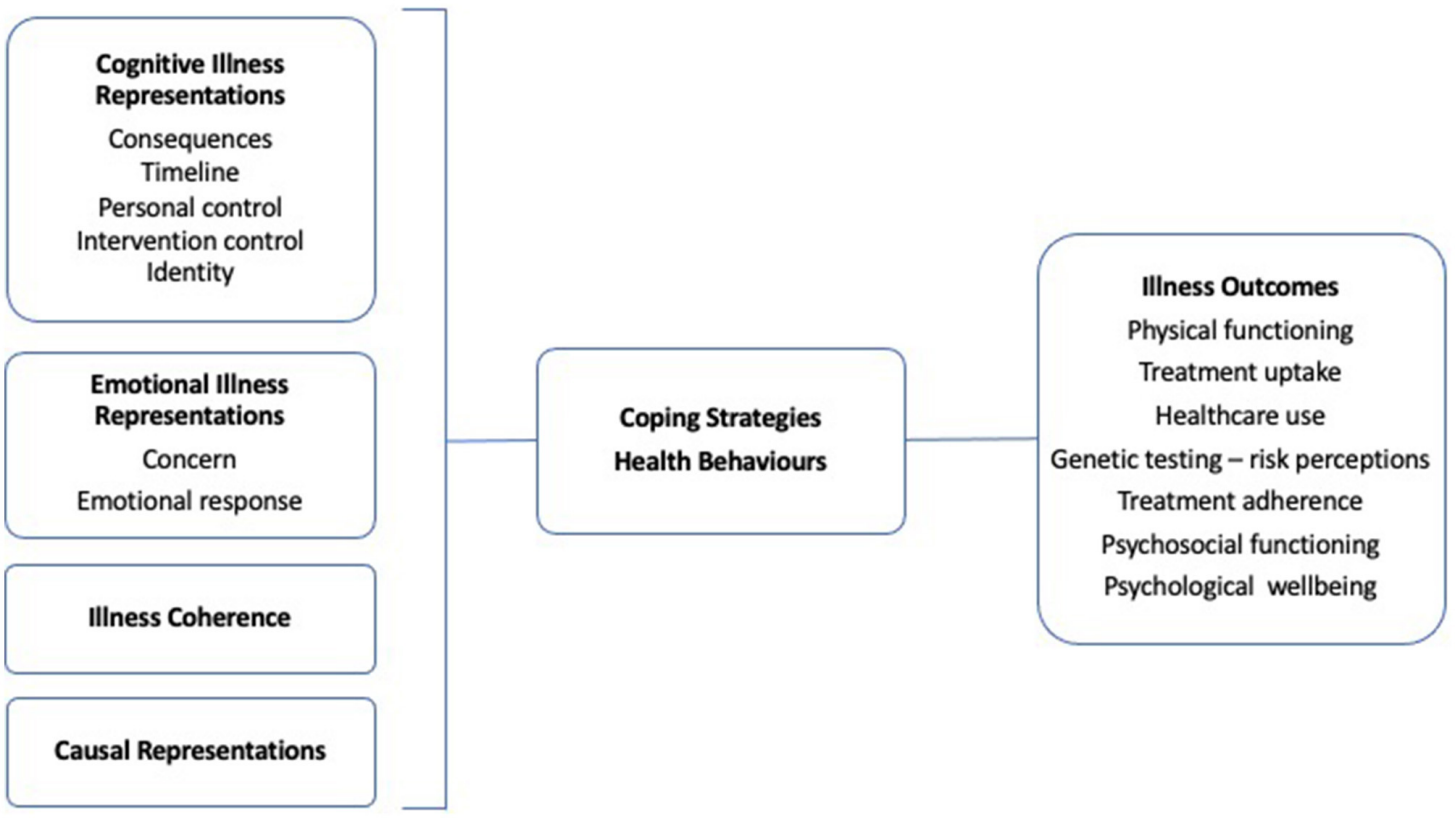
The common-sense model of self-regulation. Basic process model based on Petrie et al., 2007 (18).

The Common-Sense Model of Self-regulation (17) has been utilised to understand adjustment to illness. Illness perceptions refer to cognitive representations that patients hold about their illness. These perceptions have been shown to be important determinants of illness-related actions and behaviours and have been associated with important disease outcomes, including treatment adherence (Figure 1) (18). The five characteristic dimensions of illness representations are: Cause (beliefs about biopsychosocial factors that caused the disease); Consequences (beliefs about the overall physical and mental impact of the disease); Identity (beliefs about the disease, including attributions of symptoms to the disease); Timeline (beliefs about disease and symptom course) and Cure/controllability (beliefs about the efficacy of treatment, including coping behaviours on disease course). These dimensions are presumed to interact to influence intention-behaviour relationships and action planning, and ultimately health behaviour and outcomes (19).

To date, only one study has directly examined illness perceptions in ALS. Miglioretti et al. (20) showed that patients clustered as “non-adaptors” based on their illness perceptions had lower psychological well-being, and in turn faster disease progression (20). This is in line with previous findings showing an association between psychological status and disease outcomes, including survival time among ALS patients without dementia (21, 22). Accordingly, a focus on illness perceptions in the context of behavioural changes in ALS may offer a promising avenue to guide future ALS care best practises (11). The aims of the current study were to explore the dimensions of illness perceptions in a factor-analytic framework, to determine its relationship to behavioural change in ALS.

## Materials and Methods

### Participants

Thirty-nine patients evaluated at a specialist tertiary ALS clinic were recruited from the ForeFront ALS/FTD Clinic, Sydney. Patients met current clinical diagnostic criteria for clinically definite or probable ALS (23, 24). Diagnosis was established in line with current consensus criteria by a multidisciplinary team comprising of neurologists with expertise in ALS and FTD, psychologists, ALS clinical nurse consultant. All participants underwent comprehensive clinical and neurophysiological investigation, including cognitive and behavioural screening. Exclusion criteria included severe physical impairment, prior history of other neurological or psychiatric illness that would hinder participation, chronic alcohol or other substance abuse, and limited English proficiency.

Ethical approval was granted by the South Eastern Sydney Local Health District [11/103 (HREC/11/POWH/148); 10/126] and the University of Sydney Human Research Ethics Committees (2014/050).

### Measures

#### Physical Status

The revised version of the ALS Functional Rating Scale (ALSFRS-R) is a 12-item measure of bulbar, fine motor, gross motor and respiratory functions. Each item is rated on a four-point scale ranging from 0 (no function) to 4 (normal function) with a total score of 48 indicating normal function (25).

#### Global Cognitive Function

Global cognitive status was evaluated with the Addenbrooke's Cognitive Examination – III (ACE-III) or its predecessor (ACE-R). The ACE-III/ACE-R is a brief multi-domain cognitive assessment designed to identify early cognitive symptoms in dementia and comprises five subscales measuring the integrity of the following cognitive abilities: orientation, memory, fluency, language and visuospatial. A total score below 88/100 indicated suspected dementia (26, 27).

#### Behavioural Changes

The Motor Neuron Disease Behavioural Scale (MiND-B) is a nine-item informant-completed behavioural measure that evaluates the presence of apathy, disinhibition, and stereotypical behaviour. Each item is rated on a scale of 1 (everyday) to 4 (no changes from normal behaviour). A score of ≤33 is indicative of the presence of behavioural changes (28).

#### Illness Perceptions

The Brief Illness Perception Questionnaire (BIPQ) is a nine-item scale designed to assess patients' cognitive and emotional perceptions of their illness. Each of the item is rated on a scale from 0 to 10 except the causal question which requires an open-ended response. The first 5-items constitutes cognitive illness representations (the timeline item was omitted given the terminal nature of ALS): consequences (0 “No affect at all” – 10 “Severely affects my life”), personal control (0 “Absolutely no control” – 10 “Extreme amount of control”), treatment control (0 “Not at all” – 10 “Extremely helpful”), and identity (0 “No symptoms at all” – 10 “Many severe symptoms”). Two items reflect emotional illness perceptions: namely, concern (0 “Not at all concerned” – 10 “Extremely concerned”) and emotions (0 “Not at all affected emotionally” – 10 “Extremely affected emotionally”). Separate items evaluate illness coherence (0 “Don't understand at all” – 10 “Understand very clearly”) and causal representation (“The most important factors that you believe caused your illness”) (29).

### Statistical Analyses

Data were screened and analysed using IBM SPSS Statistics for Macintosh, Version 26.0. Descriptive statistics including normality (using the Shapiro–Wilk tests interpreted in conjunction with histograms, probability–probability plots and the values of skew and kurtosis) were examined to describe the characteristics of the sample and cheque for any gross violation of the assumptions underlying statistical tests used. Demographic and clinical differences between patients with and without behavioural changes were analysed by independent sample *t* tests or Mann–Whitney *U* tests where appropriate.

Principal Component Analysis (PCA) with Varimax rotation was used to clarify the relationship between illness perceptions and behavioural changes. Varimax rotation maximises the shared variance to ensure that results more discretely represent how data correlate with each principal component, therefore enhancing interpretability. Sampling adequacy was based on the Kaiser-Meyer-Olkin Measure of Sampling Adequacy for the overall data. Total scores for the seven items of the BIPQ were included in the analysis. Selection of components used Kaiser's criteria, whereby factors yielding an eigenvalue >1 were selected. Scree plots were examined to aid factor selections. Component loadings >0.50 were considered relevant and component scores were computed using the regression method. Logistic regression analysis was used to examine the relationships between the component scores and behavioural changes. In addition, component scores were also correlated with disease progression rate. The rate of disease progression (ΔFS) was estimated using the following formula: ΔFS = (48 - ALSFRS-R at “time of diagnosis”)/duration from onset to diagnosis (month) (30). A *p* value of < 0.05 was considered statistically significant for all tests, except for the Bonferroni corrections applied for multiple comparisons.

## Results

### Demographic and Clinical Characteristics

The ALS cohort consisted of 29 males and 10 females, ranging in age from 39 to 85 years (63.9 ± 2.0). Mean disease duration was 10.9 ± 2.4 months, and patients were mildly disabled based on the ALSFRS-R (41.4 ± 0.8). Most patients had limb- onset disease (69%). The most common behavioural symptom was apathy (14.7%) followed by disinhibition (8.8%) and stereotypy (5.9%). No significant differences in age, education, sex, disease onset, physical function, and cognitive status at time of assessment were identified between patients with and without behavioural changes. However, patients presenting with behavioural symptoms had a significantly longer disease duration compared to those without symptoms, *U* = 66.5, *z* = −2.7, *p* < 0.05 (Table 1).

**Table 1:** Demographic and clinical characteristics of the cohort stratified by behavioural change status.

	**Behavioural changes absent (*n* = 18)**	**Behavioural changes present(*n* = 16)**	***p*-value**
Age (mean, SD)	62.4 (11.9)	63.9 (11.2)	0.722
Years of education (median, IQR)	11.0 (2.6)	15.0 (6.3)	0.120
Male (n, %)	11 (32.4)	14 (41.2)	0.125^a^
Limb onset (n, %)	14 (41.2)	10 (29.4)	0.457^a^
Months since diagnosis (median, IQR)	1.5 (5.0)	9.0 (18.0)	0.007^*^
ALSFRS- R total (median, IQR)	43.0 (4.0)	42.5(8.0)	0.741
ACE total (median, IQR)	92.5 (11.0)	93.8 (11.0)	0.959

*ALSFRS-R, ALS Functional Rating Scale – Revised; IQR, Interquartile range; SD, Standard deviation. Behavioural data (MiND-B) from 34 patients only*.

a *Fisher's Exact Probability Test*.

* *p < 0.05*.

### Illness Perceptions Dimensions

While patients with behavioural changes tended to report high coherence and greater perceived consequences, symptoms and emotional responses associated with ALS, a series of Mann-Whitney U Tests revealed no significant differences in the BIPQ scores between patients with and without behavioural changes (Table 2).

**Table 2:** BIPQ scores of the cohort stratified by behavioural change status.

	**Behavioural changes absent** **(*n* = 18)**	**Behavioural changes present(*n* = 16)**	***p*-value**
BIPQ 1Consequences	7.0 (4.0)	8.5 (3.0)	0.198
BIPQ 3 Personal control	2.0 (4.0)	2.0 (4.0)	0.847
BIPQ 4 Treatment control	3.0 (4.0)	5.0 (6.0)	0.352
BIPQ 5 Identity	6.5 (4.0)	7.0 (4.0)	0.241
BIPQ 6 Concern	10.0 (3.0)	10.0 (2.0)	0.593
BIPQ 7 Coherence	8.0 (4.0)	8.5 (3.0)	0.597
BIPQ 8 Emotions	5.0 (4.0)	6.50 (5.0)	0.217

BIPQ, Brief Illness Perception Questionnaire. Behavioural data (MiND-B) from 34 patients only. Data represent median (interquartile range). *p < 0.007 (Bonferroni correction).

The factorability of the seven BIPQ items was initially examined to establish item correlations and determine whether coherent factors could be identified. Accordingly, well described criteria including the Kaiser–Myer–Olkin (KMO) measure of sample adequacy and Bartlett's test of sphericity were evaluated. The KMO measure of sampling adequacy was 0.717 and Bartlett's test of sphericity (correlations between items) was significant [χ^2^ (21) = 95.3, *p* < 0.0001], indicating that the data was suitable for PCA. Three components were extracted from the PCA (Table 3). Component 1 included the items related to illness identity, consequences, concern, emotional response which reflected the emotional impact of ALS and therefore termed “cognitive and emotion related ALS perceptions.” Component 2 consisted of items related to treatment control, personal control, which represented “cognitive- specific ALS perceptions.” Component 3 included the one item pertaining to patients' understanding about ALS (“ALS coherence”).

**Table 3:** Rotated component matrix from principal component analysis.

	**Factor 1 “Cognitive and emotion related ALS perceptions”**	**Factor 2 “Cognitive specific ALS perceptions”**	**Factor 3 “ALS coherence”**
**Eigenvalue**	**3.231**	**1.313**	**1.028**
BIPQ 5 Identity	**0.880**	−0.084	0.139
BIPQ 6 Concern	**0.858**	−0.050	−0.051
BIPQ 1Consequences	**0.834**	−0.250	−0.033
BIPQ 8 Emotions	**0.825**	−0.125	0.041
BIPQ 3 Personal control	−0.087	**0.887**	0.117
BIPQ 4 Treatment control	−0.202	**0.840**	−0.208
BIPQ 7 Coherence	0.029	−0.043	**0.986**

*ALS, Amyotrophic lateral sclerosis; BIPQ, Brief Illness Perception Questionnaire. Factor loadings ≥0.5 are highlighted in bold*.

### Illness Perceptions and Behavioural Changes

Direct logistic regression was performed to assess the impact of identified illness perception components on the likelihood that patients presented with behavioural changes. The model contained four independent variables, including disease duration (months since diagnosis), “cognitive and emotion related ALS perceptions” and “cognitive specific ALS perceptions” and “ALS coherence”. The full model containing all predictors was statistically significant, χ^2^ (4, *N* = 33) =16.5, *p* = 0.002, indicating that the model was able to distinguish between patients with and without behavioural changes. The model explained 39.4% (Cox and Snell R square) and 52.6% (Nagelkerke R squared) of the variance in behavioural changes, and correctly classified 87.9% of the cases. As shown in Table 4, only two of the independent variables made a unique statistically significant contribution to the model. The strongest predictor of presenting with behavioural changes was “cognitive and emotion-related ALS perceptions”, recording an odds ratio of 4.6 (1/0.217). This indicated that patients with behavioural changes were four times more likely to perceive greater cognitive and emotional impacts of ALS, controlling for all other predictors in the model. The odds ratio for disease duration was 1.2, indicating that for every one month following diagnosis, patients were 1.2 times more likely to present with behavioural changes, controlling for all other predictors in the model.

**Table 4:** Logistic regression predicting likelihood of the presence of behavioural changes.

							**95% C.I. for Odds Ratio**	
	**B**	**S.E**.	**Wald**	**Df**	***p*- value**	**Odds Ratio**	**Lower**	**Upper**
Disease duration	−0.19	0.08	5.40	1	0.020^*^	0.83	0.70	0.97
Factor 1 “Cognitive and emotion related ALS perceptions”	−1.53	0.65	5.46	1	0.019^*^	0.22	0.06	0.78
Factor 2 “Cognitive specific ALS perceptions”	−0.35	0.47	0.57	1	0.450	0.70	0.28	1.75
Factor 3 “ALS coherence”	0.06	0.49	0.02	1	0.897	1.07	0.41	2.77
Constant	1.65	0.76	4.74	1	0.029	5.23		

* *p < 0.05*.

### Illness Perceptions and Disease Progression Rate

Spearman's rho revealed a strong and statistically significant association between “cognitive and emotion-related ALS perceptions” and disease progression rate (rho = 0.50, *n* = 29, *p* < 0.001). Thus, the presence of greater perceived “cognitive and emotion-related ALS perceptions” was associated with faster disease progression.

## Discussion

Findings from the current study provide insight into illness perceptions in the context of behavioural changes in ALS. Three dimensions of illness perceptions were identified: cognitive and emotion related ALS perceptions; cognitive- specific ALS perceptions; and ALS coherence. The most salient dimension were the *cognitive and emotion-related ALS* perceptions, which were characterised by stronger emotional responses and beliefs regarding severity of symptoms. Patients with behavioural changes were more likely to report greater perceived *cognitive and emotion related ALS perceptions*, which in turn were associated with more rapid disease progression. Accordingly, *cognitive and emotion related ALS* perceptions may reflect more extensive disease, associated with behavioural changes, rather than natural psychological responses to a terminal diagnosis.

Of relevance, the comorbidity of ALS and FTD translate into shorter survival times for ALS-FTD patients compared to those with preserved cognitive and behavioural functioning. The presence of cognitive impairment in particular has been consistently linked to worse prognosis, which may be related to non-adherence to treatment recommendations (31–33). There is increasing evidence to suggest that behavioural changes also contribute to disease progression. In ALS patients with cognitive impairment fulfilling dementia criteria, only patients with concurrent behavioural changes had a worse prognosis (34). Similarly, more severe apathy has been linked to shorter survival compared to milder levels of apathy in ALS (5). Furthermore, increasing evidence suggests that the most common behavioural symptom of ALS, initiation apathy, involves brain regions critical for self-regulation across emotion and action domains (35, 36). Accordingly, greater perceived “cognitive and emotion related ALS perceptions” may perhaps negatively influence the initiation of health behaviours such as treatment uptake and adherence.

The current findings highlight the importance of accurate assessment of non-motor manifestations of ALS and delineation of its cognitive, emotional, and social cognitive components, which provides a basis for guiding the development of interventions. Direct assessment of patients' experiences of ALS, particularly in patients without severe cognitive impairment provides valuable clinical information to guide patient-tailored intervention. Solely relying on informant-based information concerning patients' lived experiences may be less useful in such instances (37). Similarly, the few studies conducted to date suggest significant discrepancies between patient and caregiver ratings of the psychological impact of ALS (e.g., quality of life, patient suffering, caregiver burden) (38, 39). As such, understanding a patient's illness perceptions is vital to addressing maladaptive health behaviours.

Management of behavioural symptoms forms the larger goal of preservation of psychological well-being in both patients and caregivers. Support for ALS patients presenting with behavioural symptoms and their caregivers is often recommended to assist with management of the non-motor manifestations of the disease (11). However, there have been no interventions for cognitive or behavioural symptoms systematically tried to date. General strategies to manage cognitive and behavioural symptoms based on the dementia literature have been proposed. First and foremost is patient (depending on their level of insight) and caregiver education provided by a multidisciplinary team (40). Importantly, this must be provided in the context of a rapidly progressive physical illness which is likely to restrict intervention objectives, modalities, settings, and intensity. Taken together, perhaps patient and caregiver education session initiated by the multidisciplinary team shortly after diagnosis of cognitive and behavioural involvement may be timely. Education tailored to specific “cognitive and emotion related ALS perceptions” may focus on managing extremely distressing perceptions about the impact of the ALS on functional capacity and quality of life. This may also be beneficial prior to the implementation of supportive interventions to minimise functional disability and optimise intervention uptake and adherence.

Indeed, evidence suggests that active self-care behaviours are more likely to be performed if they are perceived to be efficacious, and symptoms are believed to be somewhat controllable. On the contrary, perceiving symptoms as completely uncontrollable tends to be associated with maladaptive coping strategies such as denial and avoidance (18). Accordingly, addressing “cognitive and emotion related ALS perceptions” promptly may result in early implementation and improved adherence to supportive interventions. This is especially important in the context of cognitive and behavioural symptoms, with ALS-FTD linked to increased likelihood of non-adherence to life sustaining interventions (e.g., enteral nutrition and non-invasive ventilation) (33, 41), and would consequently require more extensive supervision and assistance on the caregivers' part.

Results from the current series of studies inevitably have implications for decision-making. ALS patients and their caregivers are often faced with the most confronting decisions of their lives, and understandably decision-making focuses on the present issues, rather than decisions about future potential care needs (42). Patient and caregivers perceived responsibilities toward each other in the context of decision-making process has been shown to be imperative (43). As such, addressing illness perceptions may provide an avenue for supported decision-making for patients and their caregivers at the outset, including an opportunity to make advanced informed decisions regarding directives for life-sustaining interventions (44).

The findings from this study should be considered in light of design and methodological limitations common to this body of research. Firstly, neuropsychological evaluation using ALS-specific instruments which were specifically developed for ALS and take into account motor impairment was not conducted [the Edinburgh Cognitive and Behavioural ALS Screen (45) and the ALS Cognitive Behavioural Screen (46)]. Thus, the use of the ACE may limit extrapolations of illness perceptions in the context of cognitive impairment. Future prospective studies using ALS-specific cognitive assessments may also clarify whether greater perceived cognitive and emotional impacts of ALS may have a role as a clinical marker that can help predict the development and progression of cognitive and behavioural symptoms. Furthermore, all ALS phenotypes were included which may have skewed disease progression rates. Relatedly, patients' illness perception ratings may have been influenced by recent clinical deterioration. The small sample size also did not allow for further subgroup analysis, particularly whether bulbar disease onset typically associated with a rapidly progressive course (47) was associated with greater perceived cognitive and emotional impacts of ALS at the outset. While bulbar, motor and respiratory functions were assessed using the ALSFRS-R, future studies incorporating more objective measures of these parameters such as speech motor performance (48), muscle strength, forced vital capacity (49) as well as adherence to recommended interventions (e.g., NIV and PEG) (33) would further clarify the relationship between illness perceptions and disease-related factors.

In conclusion, patients with behavioural changes may present with greater perceived cognitive and emotional impacts associated with ALS, which has major implications for clinical management and development of interventions and support as part of ALS care (50).

## Data Availability Statement

The raw data supporting the conclusions of this article will be made available by the authors, without undue reservation.

## Ethics Statement

The studies involving human participants were reviewed and approved by South Eastern Sydney Local Health District and the University of Sydney Human Research Ethics Committees. The patients/participants provided their written informed consent to participate in this study.

## Author Contributions

JC contributed to the conceptualisation of the study, data collection, analysis and interpretation of data, and drafting and revision of the manuscript. ED and RA contributed to data collection, interpretation of data, and revision of the manuscript. WH and MZ contributed to data collection and revision of the manuscript. MK contributed to the conceptualisation of the study, interpretation of data, revision of the manuscript, and study supervision. All authors contributed to the article and approved the submitted version.

## Funding

This study was supported by ForeFront, a large collaborative research group dedicated to the study of neurodegenerative diseases and funded by the National Health and Medical Research Council of Australia Program Grant (#1132524), Dementia Research Team Grant (#1095127), and CogSleep Centre of Research Excellence (#1152945).

## Conflict of Interest

The authors declare that the research was conducted in the absence of any commercial or financial relationships that could be construed as a potential conflict of interest.

## Publisher's Note

All claims expressed in this article are solely those of the authors and do not necessarily represent those of their affiliated organizations, or those of the publisher, the editors and the reviewers. Any product that may be evaluated in this article, or claim that may be made by its manufacturer, is not guaranteed or endorsed by the publisher.

## References

[1] Mahoney CJ Ahmed RM Huynh W Tu S Rohrer JD Bedlack RS. Pathophysiology and treatment of non-motor dysfunction in amyotrophic lateral sclerosis. CNS Drugs. (2021) 35:483–505. 10.1007/s40263-021-00820-1 33993457

[2] Montuschi A Iazzolino B Calvo A Moglia C Lopiano L Restagno G. Cognitive correlates in amyotrophic lateral sclerosis: a population-based study in Italy. J Neurol Neurosurg Psychiatry. (2015) 86:168–73. 10.1136/jnnp-2013-307223 24769471

[3] Phukan J Elamin M Bede P Jordan N Gallagher L Byrne S. The syndrome of cognitive impairment in amyotrophic lateral sclerosis: a population-based study. J Neurol Neurosurg Psychiatry. (2012) 83:102–8. 10.1136/jnnp-2011-300188 21836033

[4] Raaphorst J Beeldman E De Visser M De Haan RJ Schmand B. A systematic review of behavioural changes in motor neuron disease. Amyotroph Lateral Scler. (2012) 13:493–501. 10.3109/17482968.2012.656652 22424127

[5] Caga J Turner MR Hsieh S Ahmed RM Devenney E Ramsey E. Apathy is associated with poor prognosis in amyotrophic lateral sclerosis. Eur J Neurol. (2016) 23:891–7. 10.1111/ene.12959 26822417

[6] Kiernan MC Vucic S Cheah BC Turner MR Eisen A Hardiman O. Amyotrophic lateral sclerosis. Lancet. (2011) 377:942–55. 10.1016/S0140-6736(10)61156-7 21296405

[7] Rodriguez de Rivera FJ Oreja Guevara C Sanz Gallego I San Jose Valiente B Santiago Recuerda A Gomez Mendieta MA. Outcome of patients with amyotrophic lateral sclerosis attending in a multidisciplinary care unit. Neurologia. (2011) 26:455–60. 10.1016/j.nrleng.2011.01.010 21419529

[8] Rooney J Byrne S Heverin M Tobin K Dick A Donaghy C. A multidisciplinary clinic approach improves survival in ALS: a comparative study of ALS in Ireland and Northern Ireland. J Neurol Neurosurg Psychiatry. (2015) 86:496–501. 10.1136/jnnp-2014-309601 25550416

[9] Traynor BJ Alexander M Corr B Frost E Hardiman O. Effect of a multidisciplinary amyotrophic lateral sclerosis (ALS) clinic on ALS survival: a population based study, 1996-2000. J Neurol Neurosurg Psychiatry. (2003) 74:1258–61. 10.1136/jnnp.74.9.1258 12933930 PMC1738639

[10] Van den Berg JP Kalmijn S Lindeman E Veldink JH de Visser M Van der Graaff MM. Multidisciplinary ALS care improves quality of life in patients with ALS. Neurology. (2005) 65:1264–7. 10.1212/01.wnl.0000180717.29273.12 16247055

[11] Miller RG Jackson CE Kasarskis EJ England JD Forshew D Johnston W. Practice parameter update: the care of the patient with amyotrophic lateral sclerosis: multidisciplinary care, symptom management, and cognitive/behavioral impairment (an evidence-based review): report of the Quality Standards Subcommittee of the American Academy of Neurology. Neurology. (2009) 73:1227–33. 10.1212/WNL.0b013e3181bc01a4 19822873 PMC2764728

[12] Caga J Hsieh S Highton-Williamson E Zoing MC Ramsey E Devenney E. Apathy and its impact on patient outcome in amyotrophic lateral sclerosis. J Neurol. (2018) 265:187–93. 10.1007/s00415-017-8688-4 29189922

[13] Rabkin J Goetz R Murphy JM Factor-Litvak P Mitsumoto H Group ACS. Cognitive impairment, behavioral impairment, depression, and wish to die in an ALS cohort. Neurology. (2016) 87:1320–8. 10.1212/WNL.0000000000003035 27496520 PMC5573192

[14] Bock M Duong YN Kim A Allen I Murphy J Lomen-Hoerth C. Cognitive-behavioral changes in amyotrophic lateral sclerosis: screening prevalence and impact on patients and caregivers. Amyotroph Lateral Scler Frontotemporal Degener. (2016) 17:366–73. 10.3109/21678421.2016.1165257 27043386

[15] Chio A Vignola A Mastro E Giudici AD Iazzolino B Calvo A. Neurobehavioral symptoms in ALS are negatively related to caregivers' burden and quality of life. Eur J Neurol. (2010) 17:1298–303. 10.1111/j.1468-1331.2010.03016.x 20402747

[16] Woolley SC Moore DH Katz JS. Insight in ALS: awareness of behavioral change in patients with and without FTD. Amyotroph Lateral Scler. (2010) 11:52–6. 10.3109/17482960903171110 19714539

[17] Leventhal H ND Steele DJ. Illness representations and coping with health threats. In: Baum ATS Singer JE, editor. Handbook of Psychology and Health, Volume IV: social psychological aspects of health. Hillsdale, NJ Erlbaum (1984). p. 219–52. 10.1201/9781003044307-9

[18] Petrie KJ Jago LA Devcich DA. The role of illness perceptions in patients with medical conditions. Curr Opin Psychiatry. (2007) 20:163–7. 10.1097/YCO.0b013e328014a871 17278916

[19] Hagger MS Koch S Chatzisarantis NLD Orbell S. The common sense model of self-regulation: Meta-analysis and test of a process model. Psychol Bull. (2017) 143:1117–54. 10.1037/bul0000118 28805401

[20] Miglioretti M Mazzini L Oggioni GD Testa L Monaco F. Illness perceptions, mood and health-related quality of life in patients with amyotrophic lateral sclerosis. J Psychosom Res. (2008) 65:603–9. 10.1016/j.jpsychores.2008.05.012 19027451

[21] Johnston M Earll L Giles M McClenahan R Stevens D Morrison V. Mood as a predictor of disability and survival in patients newly diagnosed with ALS MND. Brit J Health Psych. (1999) 4:127–36. 10.1348/135910799168524

[22] Bond L Bowen G Mertens B Denson K Jordan K Vidakovic B. Associations of patient mood, modulators of quality of life, and pharmaceuticals with amyotrophic lateral sclerosis survival duration. Behav Sci. 2020 10:33. 10.3390/bs10010033 31936812 PMC7016647

[23] Brooks BR Miller RG Swash M Munsat TL. World Federation of Neurology Research Group on Motor Neuron D. El Escorial revisited: revised criteria for the diagnosis of amyotrophic lateral sclerosis. Amyotroph Lateral Scler Other Motor Neuron Disord. (2000) 1:293–9. 10.1080/146608200300079536 11464847

[24] de Carvalho M Dengler R Eisen A England JD Kaji R Kimura J. Electrodiagnostic criteria for diagnosis of ALS. Clin Neurophysiol. (2008) 119:497–503. 10.1016/j.clinph.2007.09.143 18164242

[25] Cedarbaum JM Stambler N Malta E Fuller C Hilt D Thurmond B. The ALSFRS-R: a revised ALS functional rating scale that incorporates assessments of respiratory function. BDNF ALS Study Group (Phase III). J Neurol Sci. (1999) 169:13–21. 10.1016/S0022-510X(99)00210-5 10540002

[26] Mioshi E Dawson K Mitchell J Arnold R Hodges JR. The Addenbrooke's Cognitive Examination Revised (ACE-R): a brief cognitive test battery for dementia screening. Int J Geriatr Psychiatry. (2006) 21:1078–85. 10.1002/gps.1610 16977673

[27] Hsieh S Schubert S Hoon C Mioshi E Hodges JR. Validation of the Addenbrooke's Cognitive Examination III in frontotemporal dementia and Alzheimer's disease. Dement Geriatr Cogn Disord. (2013) 36:242–50. 10.1159/000351671 23949210

[28] Mioshi E Hsieh S Caga J Ramsey E Chen K Lillo P. A novel tool to detect behavioural symptoms in ALS. Amyotroph Lateral Scler Frontotemporal Degener. (2014) 15:298–304. 10.3109/21678421.2014.896927 24863641

[29] Broadbent E Petrie KJ Main J Weinman J. The brief illness perception questionnaire. J Psychosom Res. (2006) 60:631–7. 10.1016/j.jpsychores.2005.10.020 16731240

[30] Kimura F Fujimura C Ishida S Nakajima H Furutama D Uehara H. Progression rate of ALSFRS-R at time of diagnosis predicts survival time in ALS. Neurology. (2006) 66:265–7. 10.1212/01.wnl.0000194316.91908.8a 16434671

[31] Elamin M Phukan J Bede P Jordan N Byrne S Pender N. Executive dysfunction is a negative prognostic indicator in patients with ALS without dementia. Neurology. (2011) 76:1263–9. 10.1212/WNL.0b013e318214359f 21464431

[32] Hu WT Seelaar H Josephs KA Knopman DS Boeve BF Sorenson EJ. Survival profiles of patients with frontotemporal dementia and motor neuron disease. Arch Neurol. (2009) 66:1359–64. 10.1001/archneurol.2009.253 19901167 PMC2881327

[33] Olney RK Murphy J Forshew D Garwood E Miller BL Langmore S. The effects of executive and behavioral dysfunction on the course of ALS. Neurology. (2005) 65:1774–7. 10.1212/01.wnl.0000188759.87240.8b 16344521

[34] Hu WT Shelnutt M Wilson A Yarab N Kelly C Grossman M. Behavior matters–cognitive predictors of survival in amyotrophic lateral sclerosis. PLoS ONE. (2013) 8:e57584. 10.1371/journal.pone.0057584 23460879 PMC3583832

[35] Caga J Tu S Dharmadasa T Tse NY Zoing MC Huynh W. Apathy is associated with parietal cortical-subcortical dysfunction in ALS. Cortex. (2021). 10.1016/j.cortex.2021.02.029 33867121

[36] Langner R Leiberg S Hoffstaedter F Eickhoff SB. Towards a human self-regulation system: Common and distinct neural signatures of emotional and behavioural control. Neurosci Biobehav Rev. (2018) 90:400–10. 10.1016/j.neubiorev.2018.04.022 29730485 PMC5994341

[37] Zgaljardic DJ Borod JC Foldi NS Rocco M Mattis PJ Gordon MF. Relationship between self-reported apathy and executive dysfunction in nondemented patients with Parkinson disease. Cogn Behav Neurol. (2007) 20:184–92. 10.1097/WNN.0b013e318145a6f6 17846518 PMC4456014

[38] Adelman EE Albert SM Rabkin JG Del Bene ML Tider T O'Sullivan I. Disparities in perceptions of distress and burden in ALS patients and family caregivers. Neurology. (2004) 62:1766–70. 10.1212/01.WNL.0000125180.04000.A4 15159475

[39] Trail M Nelson ND Van JN Appel SH Lai EC. A study comparing patients with amyotrophic lateral sclerosis and their caregivers on measures of quality of life, depression, and their attitudes toward treatment options. J Neurol Sci. (2003) 209:79–85. 10.1016/S0022-510X(03)00003-0 12686407

[40] Strong MJ. Amyotrophic Lateral Sclerosis and the Frontotemporal Dementias Oxford. United Kingdom: Oxford University Press (2012). 10.1093/med/9780199590674.001.0001

[41] Chio A Ilardi A Cammarosano S Moglia C Montuschi A Calvo A. Neurobehavioral dysfunction in ALS has a negative effect on outcome and use of PEG and NIV. Neurology. (2012) 78:1085–9. 10.1212/WNL.0b013e31824e8f53 22442427

[42] Hogden A Greenfield D Nugus P Kiernan MC. What influences patient decision-making in amyotrophic lateral sclerosis multidisciplinary care? A study of patient perspectives. Patient Prefer Adherence. (2012) 6:829–38. 10.2147/PPA.S37851 23226006 PMC3514070

[43] Foley G Hynes G. Decision-making among patients and their family in ALS care: a review. Amyotroph Lateral Scler Frontotemporal Degener. (2018) 19:173–93. 10.1080/21678421.2017.1353099 28799808

[44] Silverstein MD Stocking CB Antel JP Beckwith J Roos RP Siegler M. Amyotrophic lateral sclerosis and life-sustaining therapy: patients' desires for information, participation in decision making, and life-sustaining therapy. Mayo Clin Proc. (1991) 66:906–13. 10.1016/S0025-6196(12)61577-8 1921500

[45] Abrahams S Newton J Niven E Foley J Bak TH. Screening for cognition and behaviour changes in ALS. Amyotroph Lateral Scler Frontotemporal Degener. (2014) 15:9–14. 10.3109/21678421.2013.805784 23781974

[46] Woolley SC York MK Moore DH Strutt AM Murphy J Schulz PE. Detecting frontotemporal dysfunction in ALS: utility of the ALS Cognitive Behavioral Screen (ALS-CBS). Amyotroph Lateral Scler. (2010) 11:303–11. 10.3109/17482961003727954 20433413

[47] Chio A Logroscino G Hardiman O Swingler R Mitchell D Beghi E. Prognostic factors in ALS: A critical review. Amyotroph Lateral Scler. (2009) 10:310–23. 10.3109/17482960802566824 19922118 PMC3515205

[48] Green JR Yunusova Y Kuruvilla MS Wang J Pattee GL Synhorst L. Bulbar and speech motor assessment in ALS: challenges and future directions. Amyotroph Lateral Scler Frontotemporal Degener. (2013) 14:494–500. 10.3109/21678421.2013.817585 23898888 PMC3833808

[49] Paganoni S Cudkowicz M Berry JD. Outcome measures in amyotrophic lateral sclerosis clinical trials. Clin Investig. (2014) 4:605–18. 10.4155/cli.14.52 28203356 PMC5305182

[50] Kiernan MC Vucic S Talbot K McDermott CJ Hardiman O Shefner JM. Improving clinical trial outcomes in amyotrophic lateral sclerosis. Nat Rev Neurol. (2021) 17:104–18. 10.1038/s41582-020-00434-z 33340024 PMC7747476

